# Probing the Functional Mechanism of *Escherichia coli* GroEL Using Circular Permutation

**DOI:** 10.1371/journal.pone.0026462

**Published:** 2011-10-18

**Authors:** Tomohiro Mizobata, Tatsuya Uemura, Kazuhiro Isaji, Takuma Hirayama, Kunihiro Hongo, Yasushi Kawata

**Affiliations:** 1 Department of Chemistry and Biotechnology, Graduate School of Engineering, Tottori University, Tottori, Japan; 2 Department of Biotechnology, Faculty of Engineering, Tottori University, Tottori, Japan; 3 Department of Biomedical Science, Institute of Regenerative Medicine and Biofunction, Graduate School of Medical Science, Tottori University, Tottori, Japan; University of South Florida College of Medicine, United States of America

## Abstract

**Background:**

The *Escherichia coli* chaperonin GroEL subunit consists of three domains linked via two hinge regions, and each domain is responsible for a specific role in the functional mechanism. Here, we have used circular permutation to study the structural and functional characteristics of the GroEL subunit.

**Methodology/Principal Findings:**

Three soluble, partially active mutants with polypeptide ends relocated into various positions of the apical domain of GroEL were isolated and studied. The basic functional hallmarks of GroEL (ATPase and chaperoning activities) were retained in all three mutants. Certain functional characteristics, such as basal ATPase activity and ATPase inhibition by the cochaperonin GroES, differed in the mutants while at the same time, the ability to facilitate the refolding of rhodanese was roughly equal. Stopped-flow fluorescence experiments using a fluorescent variant of the circularly permuted GroEL CP376 revealed that a specific kinetic transition that reflects movements of the apical domain was missing in this mutant. This mutant also displayed several characteristics that suggested that the apical domains were behaving in an uncoordinated fashion.

**Conclusions/Significance:**

The loss of apical domain coordination and a concomitant decrease in functional ability highlights the importance of certain conformational signals that are relayed through domain interlinks in GroEL. We propose that circular permutation is a very versatile tool to probe chaperonin structure and function.

## Introduction

Stress-induced protein denaturation and aggregation in *E. coli* is mitigated by the actions of various heat shock proteins, which are mobilized in response to the initial stress stimulus. The chaperonin proteins GroEL and GroES, large, ring-like protein complexes that bind protein molecules that have denatured and are prone to aggregation, support a major portion of this stress response [1]. After binding, GroEL and GroES cooperate to encapsulate the bound protein and isolate it from the surrounding environment. The binding of the nucleotide ATP to GroEL modulates this process of encapsulation. After an interval prescribed by the hydrolysis of bound ATP by GroEL, the protein is released into solution, either in a form no longer susceptible to aggregation, or one that is ready to attempt refolding to the native state.

The mechanism of protein encapsulation and release by GroEL is supported by the structure of the GroEL subunit, which consists of three distinct domains (apical, intermediate, and equatorial) that are linked by two hinge-like regions [2], [3]. Since the polypeptide backbone of GroEL runs through the three domains according to the sequence equatorial-intermediate-apical-intermediate-equatorial [2], each hinge region is composed of two polypeptide strands oriented anti-parallel-wise. Upon ATP binding to GroEL, an intricate series of conformational changes consisting mainly of bulk domain movements change the quaternary structure of GroEL to one that encapsulates the unfolded protein molecule and binds GroES to GroEL [3]–[6]. Recent studies have been involved in elucidating precisely how the GroEL subunit modifies its form to accomplish the complex cyclic mechanism of chaperonin action [7]–[10]. These studies have indicated that the three domains of GroEL change their orientation relative to each other in a highly complex fashion, and that various conformational signals between domains are communicated through the two hinges that bind the domains [11]–[13].

We were interested in probing in more detail the relationships between the subunit structure of GroEL and its functional mechanism. To this end we reasoned that it might be possible to perturb the structure in a novel manner using circular permutation [14]. Circular permutation of proteins is achieved by linking the N- and C-termini of a certain protein with a suitable linker sequence, and then reopening this circularized sequence at a different position to "relocate" the polypeptide ends. In proteins where the original N-and C-termini are in close spatial proximity to each other in the native structure, it becomes possible to obtain circularly permuted variants that retain the activity of the original protein [15]–[18].

Circular permutation has been previously used by Trent and colleagues to obtain novel truncated versions of the thermostable Class II chaperonins for use in various nanotechnological applications [19], [20]. Examples of applying this method to Class I chaperonins, with the intent to elucidate aspects of the molecular mechanism of chaperonin function, have not been reported. We describe here our efforts to obtain and characterize various circularly permuted (CP) forms of *E. coli* GroEL. We succeeded in obtaining a number of CP GroEL mutants that partially retained the structural and functional characteristics of the wild type chaperonin, which demonstrated that the basic subunit structure of GroEL was robust and amenable to circular permutation. Also, we found that the functional characteristics of each CP mutant were different, indicating that perturbing different locations of the polypeptide backbone affected the functional mechanism in a relatively subtle manner. In more detailed experiments involving one specific CP mutant, we found that a specific conformational change involving the GroEL apical domain had been suppressed. Further experiments supported the notion of a chaperonin molecule with an apical domain that had lost its ability to act in a coordinated manner. This loss of coordination, brought about by the disruption of domain linkages, served to highlight the importance of certain conformational signals that are communicated between domains. The fact that that partial chaperonin function still remained, even when such a coordinated molecular mechanism was compromised, demonstrated the basic robustness of the GroEL functional mechanism.

## Results

### Structure and purification of CP GroEL

**Figure 1:**
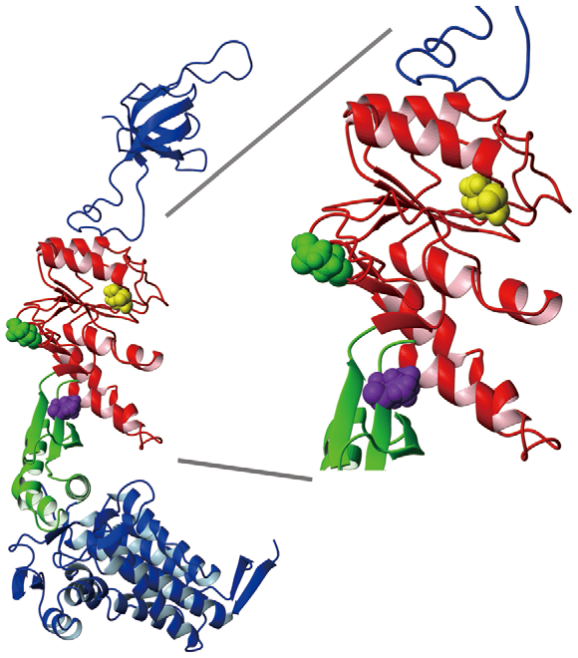
Locations of the N- and C-termini in the three circular permutation mutants of the present study. An X-ray structural model of a single GroEL/GroES subunit pair taken from PDB structure file 1 AON [3], depicting the three locations where the N- and C-termini were relocated in the three CP mutants. The locations are indicated by colored CPK representation of the first (N-terminal end) amino acid of each CP mutant, excepting the starting methionine and any extraneous amino acids that were added as a consequence of the experimental protocol (see Materials and Methods). The *green* molecule denotes Glu 209, the *yellow* molecule Val 254, and the *magenta* molecule Val 376 in wild type GroEL.

Figure 1 shows the structure of GroEL subunit with space-filled residues that indicate the positions of the newly introduced polypeptide termini in the CP mutants [3]. Our goal in this study was to obtain active, circularly permuted variants of the GroEL subunit to probe the relationship between subunit structure and the GroEL functional mechanism (with a particular interest in interdomain communications). We constructed and studied a total of 3 CP GroEL variants. We designed the CP209 mutant (polypeptide ends located at the position shown in *green*) as an initial feasibility study by selecting a site in the GroEL subunit that would be relatively tolerant to circular permutation (*i. e*., not located within clearly defined alpha helices or beta sheets). The CP254 (*yellow*) and CP376 (*magenta*) mutants were selected from a pool of randomly constructed candidates according to their relatively high expression in *E. coli* supernatant. The newly introduced polypeptide ends of CP254 were located in a region of the apical domain involved in the binding of unfolded protein and GroES to the GroEL apical domain. In CP376, the polypeptide ends were located within Hinge 2, which connects the apical and intermediate domains [3]. Of these three mutants, the CP376 mutant satisfied the greatest number of criteria set forth initially in our experimental goals.

Table 1 details the N- and C-terminal amino acid sequences of all of the CP GroEL mutants used in the present study, and compares them with the corresponding native sequence of GroEL. CP209, having been designed specifically, does not possess any amino acids other than those contained in the native GroEL sequence. In contrast, both CP254 and CP376 possess short stretches of sequence at both the N-terminal and C-terminal ends that are not of the native sequence. In the present study, we elected to proceed with initial mutant characterization without removing these extra residues because preliminary efforts showed that the simple removal of these extraneous sequences resulted in protein precipitation. However, in the case of CP376, a certain degree of polypeptide end optimization was undertaken successfully to obtain a more highly expressed variant of CP376. This variant was then used to construct CP376-RW, which possesses a fluorescent tryptophan residue in its apical domain. This derivative mutant was used in subsequent experiments.

**Table 1:** N- and C- terminal sequences of the circularly permuted GroEL subunits constructed in this study.

Mutant	Corresponding sequence in WT	C-terminal sequence	N-terminal sequence
CP209	^206^ Asn-Lys-Pro- 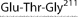	Asn-Lys-Pro ^C^	^N^ *Met*- 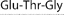
CP254	^251^ Ala-Glu-Asp- 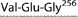	Ala-Glu-Asp *-Val-Ala-Asn* ^C^	^N^ *Met-Ala-Ala-Ala-Gln-Tyr-* 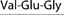
CP376	^372^ Lys-Leu-Gly-Gly- 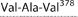	Lys-Leu-Gly *-Ala-Asn* ^C^	^N^ *Met-Ala-Gly-Gly-Ala-* 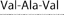
CP376-RW	^372^ Lys-Leu-Gly-Gly- 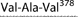	Lys-Leu-Gly-*Ala-Asn* ^C^	^N^ *Met-Gly-Ala*- 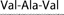

Underlines and <$>\scale 90%\raster="rg1"<$>wavy underlines are added to highlight corresponding sequence elements between each CP mutant and wild type (WT). Sequences in *italics* denote amino acids that are not from the native sequence of GroEL, added as a consequence of the circular permutation protocol. In addition, we found that in CP376, the N-terminal amino acid sequence had been altered, and apparently insertion of an extra alanine residue had occurred (double underlined). We decided to name this mutant CP376 based on the fact that the native GroEL amino acid sequence continues uninterrupted after Val376. GroEL CP376-RW contains an additional Arg to Trp point mutation at the position corresponding to Arg231 in the wild type sequence.

Each mutant was expressed in *E. coli* and purified according to the protocol used for wild type GroEL, and no significant changes in protocol were necessary to purify each protein to homogeneity. In each case, gel filtration profiles observed during purification indicated that the quaternary structure of each CP variant was similar to the tetradecameric structure of wild type GroEL (data not shown).

**Figure 2:**
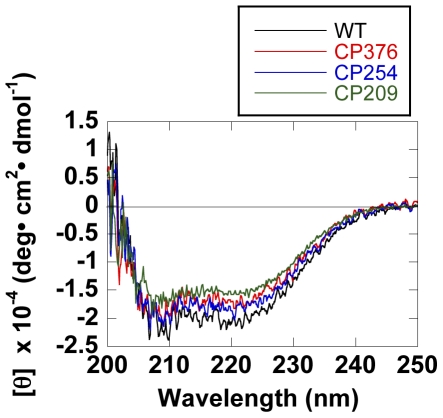
CD spectra of wild type and CP GroELs. Spectra were recorded at 25°C.

Figure 2 shows the CD spectra for CP209, CP254, and CP376 GroEL, comparing the secondary structural content of these CP mutants with wild type. The spectra were fundamentally similar in shape, with slight differences in mean residue ellipticities. The secondary structures of these three mutants were fundamentally similar to that of wild type GroEL.

### Functional characteristics

**Figure 3:**
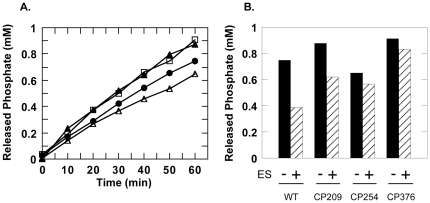
ATPase activities of wild type and CP GroELs. *A.* Basal ATPase activities measured at 25°C. *Closed circles* indicate wild type GroEL, *closed triangles* indicate CP209. *Open triangles* indicate CP254 and *open squares* indicate CP376 ATPase activities. *B.* ATPase activities in the absence and presence of an equimolar concentration of GroES heptamer. *Columns* indicate the concentration of inorganic phosphate that was released after a 60 min incubation at 25°C. Addition of GroES is indicated below each column pair (*hatched columns*). *Labels* below each column denote the chaperonin measured.

Figure 3 shows the ATPase activities of CP209, CP254 and CP376 in the presence of saturating concentrations of ATP. The ATPase activities of the three CP mutants could be summarized as follows: CP209 and CP376 displayed a higher basal ATPase activity relative to wild type, while CP254 displayed a net decrease in basal ATPase activity. Furthermore, while the ATPase activity of CP209 was sensitive to GroES and suppressed by ∼25% upon addition of an equimolar concentration this co-chaperonin, the ATPase activities of the other two mutants were relatively indifferent. Keeping in mind that all three mutants have their polypeptide ends relocated to the apical domain region and therefore relatively distant from the ATPase active site, the varied effects of each mutation on the GroEL ATPase activity reflects a delicate control of this activity by various structural elements in the apical domain.

**Figure 4:**
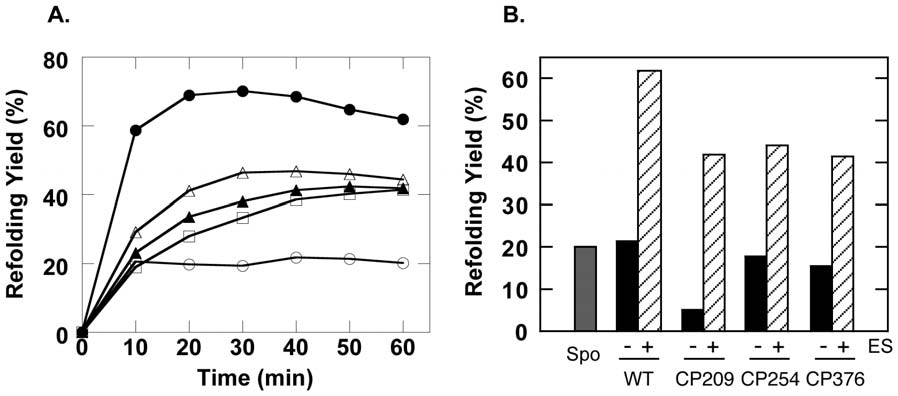
Refolding assays of bovine rhodanese performed under non-permissive conditions at 25°C. *A.* Refolding profiles of rhodanese in the presence of GroES, ATP and wild type/CP GroEL. *Open circles* indicate spontaneous refolding, *closed circles* indicate refolding in the presence of wild type GroEL. The other traces were measured in the presence of the following CP mutants: *closed triangles*, CP209, *open triangles*, CP254, *open squares*, CP376. *B.* The refolding of rhodanese was a GroES-dependent event. Refolding yields after a 60 min incubation at 25°C are shown. The *grey* column indicates spontaneous yields, and for each chaperonin, the *solid* column denotes refolding yields in the absence of GroES and the *hatched* column indicates yield in the presence of equimolar GroES.

Figure 4 shows the abilities of these three CP mutants to assist the folding of bovine rhodanese. Here, it is interesting to note that all three CP mutants were capable of assisting the refolding of rhodanese to roughly the same extent, with yields that were lower than the wild type, but nevertheless significantly improved relative to spontaneous yields. Also, it should be noted that with regard to CP254 and CP376, we observed that the lack of a response to GroES in the ATPase assays (Figure 3B) was not due to an inability to bind the co-chaperonin, since refolding of rhodanese was GroES-dependent for these mutants (Figure 4B). In these two latter circular permutation variants, the ATPase inhibitory activities have been decoupled from substrate protein release and folding assistance abilities.

From these initial characterizations we concluded that the subunit structure of GroEL was fundamentally robust and receptive to analysis by circular permutation. We next decided to characterize further the structural and functional characteristics of CP376, the mutant in which a crucial interdomain linkage had been perturbed.

### Dynamic conformational changes in the apical domain of GroEL CP376

The polypeptide termini of CP376 had been relocated to a site that was situated within Hinge 2, which links the apical and intermediate domains of GroEL and is considered to be an important site through various signals between domains are communicated. To probe the effects of this circular permutation in more detail, we introduced a fluorescent tryptophan residue to monitor the dynamic movements of the CP376 apical domain. We replaced an arginine residue (Arg 231) with a fluorescent tryptophan, at a location that we had previously used to characterize conformational changes in the wild type molecule (GroEL R231W, [6]). In the process we also optimized the expression of this mutant in *E. coli* by removing the first two amino acids from the N-terminus (*c. f.* Materials and Methods).

**Figure 5:**
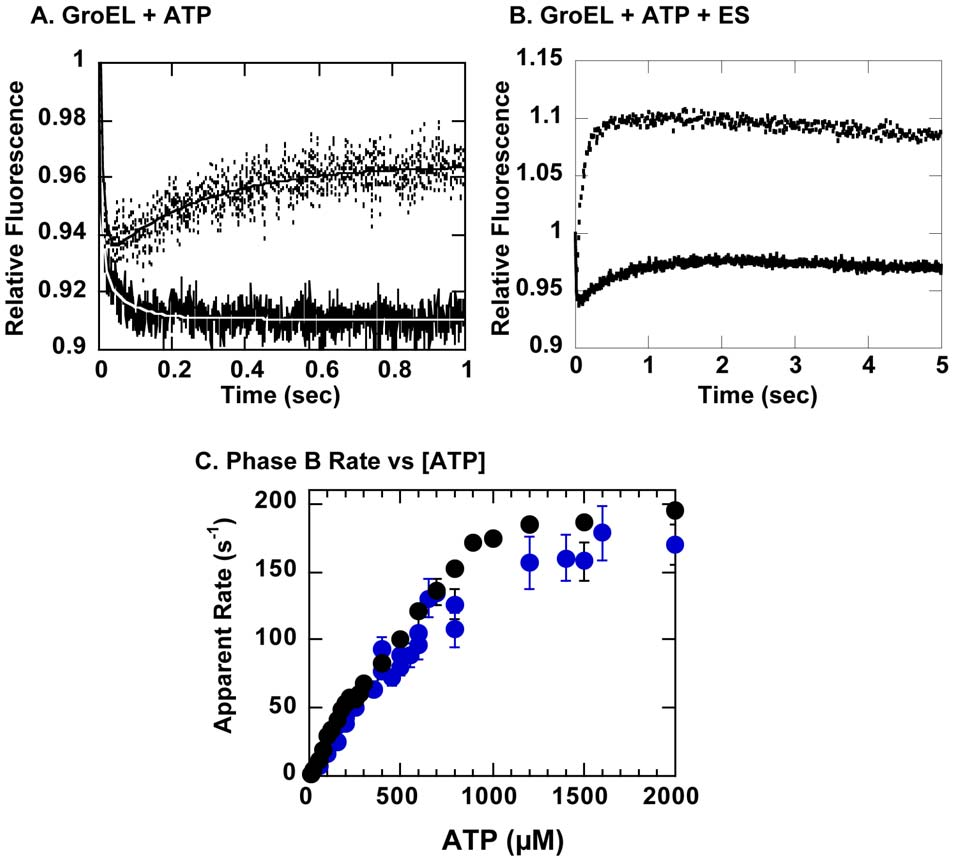
Stopped-flow fluorescence analysis of GroEL R231W and GroEL CP376-RW. Experiments were performed at 25°C. Solid traces indicate changes in tryptophan fluorescence for CP376-RW and dotted traces indicate fluorescence changes for GroEL R231W. *A.* Changes triggered by addition of 1 mM ATP, *B.* changes triggered by addition of 1 mM ATP and an equimolar concentration of GroES heptamer. In *A.*, fits to the raw traces are also shown; in white for CP376-RW and in black for R231W. The kinetic constants derived from the fits are as follows: for CP376-RW; *k*_1_ = 145.8±13.2 s^−1^, Amp_1_ = 0.086±0.004, *k*_2_ = 14.2±2.0 s^−1^, Amp_2_ = 0.017±0.002. For R231W; *k*_1_ = 116.4±7.9 s^−1^, Amp_1_ = 0.076±0.003, *k*_2_ = 2.1±0.15 s^−1^, Amp_2_ = −0.037±0.0008. Values are shown as mean ± standard errors. Negative values for amplitude denote phases with increases in fluorescence. *C.* The changes in the rate constant of Phase B in GroEL CP376-RW as a function of the ATP concentration. Kinetic traces were measured under conditions identical to that for Figure 5A and varying the concentration of ATP during measurement. The traces were analyzed to obtain the value of *k* (± standard error) at each ATP concentration. The results of two separate experimental sessions are shown (*blue filled circles*). For comparison, results of a previous identical experiment performed on GroEL R231W [6] is shown in *black circles*.

When we performed stopped-flow fluorescence experiments on the resultant fluorescent mutant CP376-RW, we found that CP376-RW behaved essentially in a manner similar to the wild type-like protein (GroEL R231W) upon addition of ATP (Figure 5A), with a single significant exception. When compared with GroEL R231W, we observed that in CP376-RW, the fluorescence increase corresponding to Phase C, referenced in Taniguchi *et al.* [6] and Yoshimi *et al.* [21], was missing. In its place we observed a relatively rapid fluorescence decrease (*k*_app_ = 14 s^−1^) with a relative amplitude that was ∼10% of the amplitude for Phase B. The characteristics of other observable phases, A, B, D, were affected only minimally in this mutant. However, when we performed stopped-flow fluorescence experiments by adding ATP and GroES (Figure 5B), we found that Phase S, the kinetic transition attributed to rearrangements caused by GroES binding, was also greatly affected in both relative amplitude and apparent rates.

Since the effects of circular permutation seemed to be localized very specifically to Phase C (and S) with minimal effects on the other kinetic transitions (Figure 5A), we next set out to see if this was indeed the case by probing certain kinetic characteristics of CP376-RW in more detail. Figure 5C shows the rate constant of Phase B of GroEL CP376-RW as a function of the ATP concentration. As outlined in Taniguchi *et al*. [6] and shown for reference in Figure 5C (*black circles*), the rate constant of Phase B in GroEL R231W increases in a bisigmoidal manner as the concentration of ATP is increased. The apparent rate constant of Phase B in CP376-RW increased in a similar manner upon increased ATP concentration (Figure 5C, *blue circles*). The rate constants were virtually identical at low ATP concentrations, and slightly smaller values were seen at ATP concentrations greater than 500 µM. The quality of the data at present is too poor to determine if the Phase B rate constant in GroEL CP376-RW was also bisigmoidally dependent on the ATP concentration; however, overall, the behavior of Phase B in CP376-RW seems to closely resemble that in GroEL R231W.

### Electron microscopy of GroEL CP376

**Figure 6:**
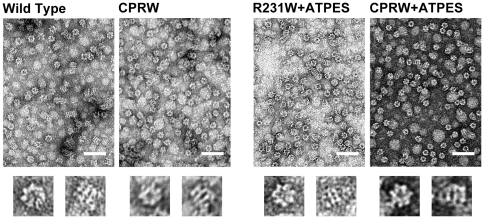
Electron Micrographs of GroEL CP376-RW. Samples were stained with 1% uranyl acetate. The scale bar in each figure indicates 100 nm. Magnification was x 60,000. *Wild Type* denotes wild type GroEL, *R231W* indicates GroEL R231W. *CPRW* denotes samples of GroEL CP376-RW, and +*ATPES* indicates samples where 0.2 mM ATP and an equimolar concentration of GroES were added in the course of sample preparation (see Materials and Methods). Below each panel, representative zoomed images of an end-on view (left) and side view (right) particle for each sample are shown.

In order to obtain structural data relevant to our understanding of the changes in subunit conformational dynamics observed in Figure 5, we monitored the structure of GroEL CP376-RW (Figure 6) under various conditions using electron microscopy.

Negatively stained images of GroEL CP376-RW (Figure 6; *CPRW*) showed that CP376-RW retained the double-heptameric ring structure of wild type GroEL (Figure 6; *Wild Type*; compare zoomed views below each panel), in agreement with the gel filtration elution profiles that we observed during purification. Using negative staining, it was difficult to distinguish any structural elements that differed from wild type GroEL. We next prepared GroEL-ADP-GroES complexes by adding 0.2 mM ATP and an equimolar concentration of GroES to each GroEL and incubated this sample for 2 h at 37°C. Under these conditions the ATP added would be hydrolyzed exhaustively, and in wild type GroEL samples, the chaperonin would form a stable GroEL-ADP-GroES bullet complex. As seen in Figure 6, we confirmed that this was indeed the case for GroEL R231W, which displays wild type like characteristics (*R231W+ATPES*). However, when we performed identical experiments using the GroEL CP376-RW mutant, the micrographs showed a distinct lack of bullet oligomeric forms (Figure 6; *CPRW+ATPES*). The micrograph was populated predominantly with end-on views that showed that the heptameric ring of GroEL CP376-RW was intact after hydrolysis, but the few examples of side views that we did observe all showed a squat, apo-like GroEL form.

### Complexes of GroEL CP376-RW and GroES are relatively unstable, and are formed even in the absence of ATP

Was the conspicuous lack of GroEL-GroES complexes in GroEL CP376-RW electron micrographs due to an inherent instability of this complex compared to GroEL R231W? To address this question, we set out to estimate roughly the amount of free GroES that was present under the conditions that we used in preparing the electron microscopy samples. The samples were separated by centrifugal concentration in a manner similar to that used in Taguchi *et al*. [22] to isolate BeFx-induced GroEL-GroES football complexes, where free proteins with a *M*_r_ less than 100 kDa would be collected in the filtrate fraction of the centrifugal concentrator.

**Figure 7:**
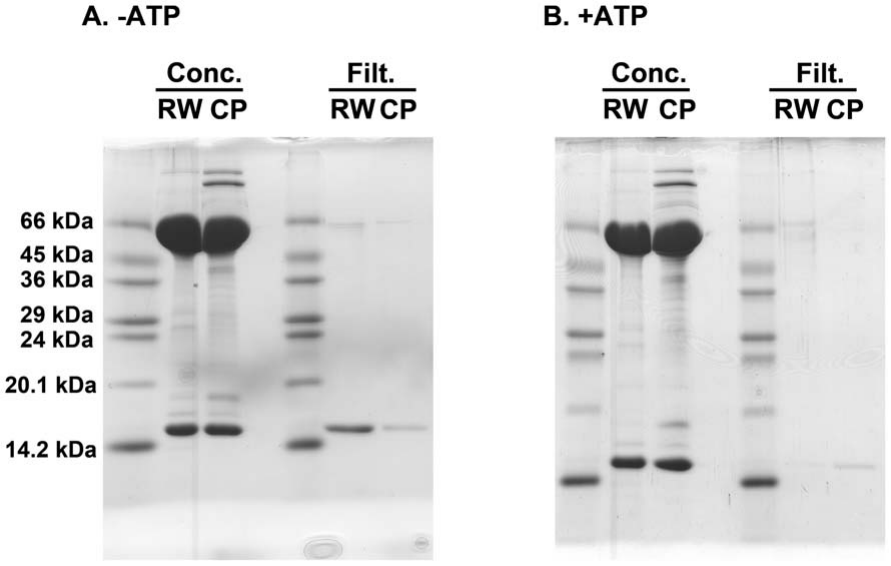
Relative stability of the GroEL-ADP-GroES complexes formed at 37°C. Each GroEL sample was incubated for 2 h at 37°C with an equimolar concentration of GroES. After incubation, samples were partitioned by centrifugal concentration (100 kDa cutoff). Samples denoted "Conc." indicate a 24 µg aliquot of each sample recovered from the upper reservoir concentrate of the filtering apparatus. Samples denoted "Filt." represent proteins that were recovered from a 200 µl aliquot of the lower reservoir filtrate of the apparatus. Experiments were performed in the absence (*−ATP, A*) and presence (*+ATP, B*) of 0.2 mM ATP. *RW* indicates GroEL R231W, and *CP* indicates GroEL CP376-RW samples. The apparent molecular weights of a commercial marker mixture (Dalton VII molecular weight marker, Sigma) are indicated to the left of the figure.

Figure 7 shows the results of this experiment, where samples were prepared in the presence and absence of 0.2 mM ATP, respectively. As seen in the figure, in the case of GroEL R231W we observed a significant amount of 70 kDa GroES protein recovered in the filtrate when ATP was omitted from the mixture, and this GroES disappeared from the filtrate when 0.2 mM ATP was added. This demonstrated a clean ATP-dependent retention of GroES in the concentrate due to stable complex formation between GroEL R231W and GroES. In contrast, when GroEL CP376-RW was incubated with equimolar GroES in the presence and absence of ATP, we noted two interesting differences in the results. First, we detected a faint band corresponding to GroES in the filtrate samples obtained in the presence of ATP, which suggested that the complex between GroEL CP376-RW and GroES was marginally less stable than the complex formed between GroEL R231W and GroES under identical conditions. Secondly, we saw that the amount of GroES recovered in the filtrate in samples incubated in the absence of ATP was significantly less than the amount of GroES recovered from the GroEL R231W samples. Considering that the experimental conditions were maintained as consistently as possible for these two samples, we believe that the apparent affinity between GroEL CP376-RW and GroES in the absence of nucleotide is significant. In other words, GroEL CP376-RW is capable of binding GroES to a certain extent in the absence of ATP.

### Incomplete encapsulation of rhodanese by CP376-RW

**Figure 8:**
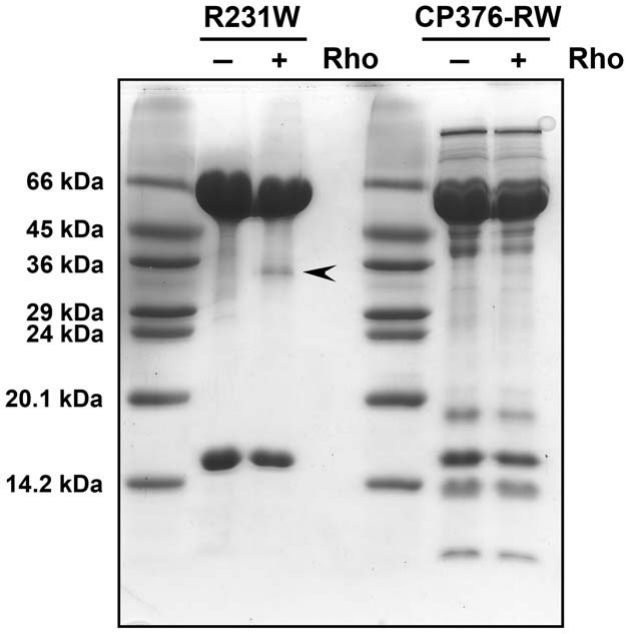
Proteinase K digestion of the BeFx-induced football complexes of GroEL R231W and GroEL CP376. Arrested football complexes of GroEL R231W and GroEL CP376-RW were prepared and incubated at 25°C. The figure shows a 15% SDS-PAGE gel of samples concentrated and recovered after a 30 min digestion with 1 µg/ml Proteinase K at 25°C. The apparent molecular weights of a commercial marker mixture (Dalton VII molecular weight marker, Sigma) are indicated to the left of the figure. *Rho* denotes unfolded rhodanese, with plus and minus tags indicating whether this unfolded protein was allowed to bind to each chaperonin prior to construction of the football complex. The *arrowhead* indicates the position of recovered rhodanese.

Wild type GroEL forms a protease-resistant symmetric football complex in the presence of ATP and BeFx that can protect unfolded protein molecules such as rhodanese from digestion [22]. In order to determine if this ability was retained in CP376-RW, we performed Proteinase K digestion experiments of the rhodanese-GroEL-GroES football complex that is formed in the presence of ATP and BeFx. Football complexes of rhodanese, GroES, and either GroEL R231W or CP376-RW GroEL were formed in the presence of excess concentrations of ATP and BeFx. Complexes were next isolated by centrifugal concentration and then subjected to a short digestion by Proteinase K. As seen in Figure 8, football complexes formed by GroEL R231W under these conditions were relatively resistant to Proteinase K digestion. The football complex was capable of protecting unfolded rhodanese molecules that bound to GroEL R231W from a Gdn-HCl unfolded state (Figure 8, *arrowhead*). When we performed identical experiments using CP376-RW, however, we found that CP376-RW was slightly more sensitive to Proteinase K digestion, judging by the significant amounts of digested product observed. What was more interesting, however, was that the rhodanese added had been completely digested under these conditions, in stark contrast to the results seen for GroEL R231W. GroEL CP376-RW has lost the ability to protect unfolded rhodanese molecules from Proteinase K digestion, which suggested that rhodanese was exposed to solvent in the putatively encapsulated state formed by GroEL CP376-RW.

## Discussion

In this study we set out to determine if circular permutation could be applied to *E. coli* GroEL to elucidate various details of its molecular mechanism. Circular permutation has been previously used to probe the relationship between the folding abilities of an amino acid sequence and its sequence topology, or to generate newly robust protein architectures that are useful in various applications [15]–[18]. A Class II member of the chaperonins, the thermosome, has been studied in the latter manner for application in various nanotechnological applications, for example [19], [20]. The general consensus seems to be that circular permutation may be applied in a large number of cases to probe protein structure and function [14].

Through a combination of both site-directed and random gene manipulation, we were able to construct and characterize a total of three circular permutants of GroEL. All three mutants maintained their quaternary structure, as observed by gel filtration during purification (not shown; see however the electron micrographs in Figure 6 for CP376-RW), and also their secondary structures were closely similar, as estimated from CD spectra (Figure 2). Functionally, all of the mutants that we characterized retained the characteristics of wild type GroEL to differing degrees. We considered it worthwhile to attempt a correlation between these differing functional characteristics and the specific position of the newly introduced polypeptide ends, to obtain new data on the structural basis of GroEL function.

### Differences and similarities in the functional characteristics of CP GroELs

As shown in Figure 3, the greatest functional disparities among the three CP mutants that we characterized were seen in the ATPase activities. Effects such as desensitization to GroES binding and both increases and decreases in basal ATPase activity all reflect the fact that the ATPase of GroEL is a highly cooperative phenomenon [23] involving various intersubunit interactions [24], and that circular permutation was able to perturb this mechanism in various ways. These differences in ATPase activity may be caused by the perturbation of various interactions, both intersubunit and intrasubunit, between certain amino acids in GroEL. Alternatively, they may be due to changes in the propagation of structural signals through the polypeptide backbone of the GroEL subunit, which would be disrupted by circular permutation. Although characterization of many more CP mutants would be necessary to determine which of these explanations is true for each case, such analyses may provide many new interesting details regarding signal communication and propagation within GroEL.

One possible way in which additional data could be obtained using the present CP mutants would be to probe if certain point mutations that identified certain regions of GroEL to be functionally important have an equal effect in both the wild type and CP mutants. By selecting a number of individual amino acid residues that have been identified in previous studies and comparing the relative effect of a point mutation of this residue in wild type and various CP GroEL mutants, it should be possible to differentiate between spatial molecular interactions and the propagation of signals through the polypeptide backbone. A very interesting topic in this context would be the recently reported theoretical study of Tehver *et al.* [25], which proposed that various amino acid residues formed an allosteric communication network within the GroEL subunit. We intend to explore this possibility vigorously in future experiments.

Despite the differences observed in the ATPase assays (Figure 3) however, we found that the three CP GroEL variants were surprisingly similar in their abilities to assist the folding of guanidine-unfolded rhodanese (Figure 4). The recovery of rhodanese remained dependent on the presence of GroES, which was interesting considering that the ATPase results demonstrated desensitization to the cochaperonin. If we consider that the suppression of GroEL ATPase activity by GroES reflects an effect of this cochaperonin to coordinate the ATPase activity of GroEL [26], [27], it seems that GroEL is capable of facilitating the folding of substrate proteins to a certain extent even when this coordination is impaired. This "residual" chaperoning activity that is retained may provide hints to a very elementary mechanism of chaperonin-facilitated protein folding *in vitro*, which would be relevant to understanding this phenomenon in better detail. More detailed experiments with a specific CP mutant, CP376, provided additional data to elaborate upon this idea.

### An uncoordinated apical domain of GroEL CP376, and a minimal chaperonin mechanism

In GroEL CP376, a prominent link between the apical and intermediate domains was disrupted. Based on the modular architecture of the GroEL subunit [3], as well as results from previous studies suggesting that the apical domain could be isolated as an autonomously active unit [28], [29] (suggestive of a relatively localized and stable protein fold), we surmised that in CP376, the mutation may have caused alterations in various apical domain conformational changes that are triggered by ATP binding, *without* destabilizing the apical domain fold significantly. Indeed, many of our experiments performed on a fluorescent variant of GroEL CP376, GroEL CP376-RW, strongly suggested that this was indeed the case with GroEL CP376, leading to both dynamic and static structural consequences that affected the chaperonin mechanism.

Numerous physicochemical studies have been performed to elucidate the dynamic characteristics of GroEL at a molecular level, and these studies have shown that in order to assist the refolding of various proteins, the GroEL subunit dramatically changes its molecular conformation in response to ATP and GroES binding. Various groups have performed detailed stopped-flow fluorescence studies that detected at least five distinct kinetic transitions in GroEL that are triggered by ATP and GroES binding [4]–[6]. Due to this extremely complex behavior, it has been difficult to assign a conclusive mechanistic explanation for each of these kinetic transitions, and possible explanations for each event have been deduced mainly from changes in rate constants and amplitude that are observed in response to changes in experimental conditions such as ATP concentration and the presence of unfolded protein molecules. For example, in a previous study, Motojima *et al*., using an ingeniously constructed GroEL mutant that could be used to estimate the relative distance between the GroEL apical and equatorial domains using FRET, characterized a kinetic transition in GroEL that was sensitive to the presence of bound unfolded protein, and from this deduced that a transition with an apparent rate of 2.7 s^−1^ represented the movement of the apical domain upwards to form an encapsulation complex [30]. We found in our own experiments using GroEL R231W a kinetic transition (Phase C) whose rate and amplitude were likewise sensitive to the presence of bound, unfolded MDH [21]. These results suggest that a series of dynamic conformational changes involving the apical domain (among them the transition characterized by Motojima *et al.* and the Phase C transition reported by us) are closely involved in the process of encapsulating unfolded protein molecules that were bound to the GroEL apical domain.

In our stopped-flow experiments, we observed that Phase C, originally characterized as above, was specifically missing from the kinetic trace displayed by GroEL CP376-RW (Figure 5A). Electron micrographs showed that the overall structure of GroEL CP376-RW was similar to wild type GroEL, with no significant differences apparent in the apical domain region (Figure 6). Our data suggested that in CP376-RW, disruption of Hinge 2 had caused the apical domain of GroEL to behave in an uncoordinated fashion, and as a consequence, a bulk conformational change of the apical domain centered on Hinge 2 has become impossible to complete. In agreement with this, we also observed that Phase S, a fluorescence signal change that is proposed to reflect conformational changes induced by GroES binding [21], was also disrupted in GroEL CP376-RW (Figure 5B). In contrast, however, the effects of the mutation on other kinetic transitions such as Phase B were minimal (Figure 5C).

A GroEL heptamer with uncoordinated apical domains would be expected to display certain characteristics consistent with such a radical perturbation. One such characteristic would involve the binding mechanism of GroES in response to ATP binding to GroEL. Since the binding of GroES to GroEL is strictly controlled by ATP binding [27], it is expected that an uncoordinated apical domain would alter this behavior considerably. Our results shown in Figures 7 and 8 are in agreement with these expectations. The GroEL CP376-RW:GroES complex was slightly less stable than a corresponding complex formed by GroEL R231W (Figure 7). More interestingly, the ATP-dependent release of GroES from GroEL CP376-RW was also less strictly enforced compared to the GroEL R231W variant; significant amounts of GroES bound to GroEL CP376-RW in the absence of ATP and were retained in the concentrate after centrifugal concentration. This indicated that the interactions between GroEL CP376-RW and GroES were persistent, and comparatively insensitive to the presence of ATP and its hydrolysis. This general trend continued in the presence of unfolded rhodanese molecules, since CP376-RW could not completely segregate proteins such as rhodanese from solution (Figure 8). To summarize, the disruption of the hinge linking the apical and intermediate domains of GroEL resulted in a chaperonin whose dynamic (Figure 5) and functional (Figures 7 and 8) characteristics were consistent with a loss of coordination in apical domain movement and a disrupted encapsulation mechanism.

Flexible, disorderly apical domain movements and a resulting inability to completely encapsulate refolding protein would also manifest itself in refolding assays, conceivably causing a loss of chaperoning activity. It was notable therefore that we could observe a net improvement in refolding yield in *in vitro* refolding assays of rhodanese (Figure 4). It is interesting to consider the refolding assistance abilities of GroEL CP376-RW and compare them with the abilities of other, partially active GroEL mutants characterized to date. For example, GroEL T89W is an example of a "rigid" mutant chaperonin that was incapable of ATP hydrolysis but nevertheless was able to assist the folding of lactate dehydrogenase in an ATP dependent manner [31]. GroEL T89W was judged to be rigid based upon its inability to hydrolyze ATP and to bind GroES. Nevertheless, an extremely rapid kinetic transition was detected immediately after ATP binding to GroEL T89W and this transition was proposed to be responsible for modulating the binding affinity of GroEL toward unfolded lactate dehydrogenase and allowing its subsequent refolding. In another example, GroEL G192W is a mutant form of GroEL with a bulky tryptophan residue introduced into Hinge 2 of the GroEL subunit. This mutant shows a relatively uncommon ability to strongly bind GroES in the absence of ATP [13]. In GroEL G192W, we observed through electron microscopy that the apical domain of one ring had been tilted into a conformation resembling the open conformation, and this was most likely allowing GroES binding in the absence of nucleotide.

Through the comparison of these functionally impaired mutant chaperonins and the characteristics exhibited by GroEL CP376 (and CP376-RW) in the present study, we observe a spectrum of static and dynamic conformations that the GroEL apical domain utilizes to alter its affinities toward unfolded polypeptide and cochaperonin GroES. The wild type subunit strongly binds unfolded protein molecules in the absence of ATP, and this affinity is shifted toward binding to GroES in the presence of nucleotide. Binding of ATP also causes a decrease in affinity toward unfolded protein that allows encapsulation by GroEL. This shift in affinity is greatly constricted in GroEL T89W, leading to a situation where release of lactate dehydrogenase is possible but binding of GroES is not. In the case of GroEL G192W, the apical domain had been locked into a conformation with a very high affinity for GroES. And in the present study, we postulate that due to the uncoordinated manner in which the apical domains of CP376 behave, this mutant is unable to modulate its affinities toward unfolded rhodanese and GroES in a manner resembling wild type GroEL. However, even under such chaotic conditions, the binding of ATP to GroEL channeled the overall reaction toward constructive assistance of the rhodanese refolding reaction. There must be a very basic molecular mechanism remaining in this mutant that couples ATP binding with rhodanese recovery. A possible minimal mechanism may simply consist of a competition between rhodanese and GroES for binding sites on the GroEL ring, which would allow a passive buffering effect of unfolded proteins to be produced, leading to the improvements in refolding yield seen in Figure 4. In this case, an explanation for the directionality toward rhodanese release and recovery upon ATP binding needs to be found. In this context, we believe that it is worthwhile to emphasize again that the effects of circular permutation and disruption of Hinge 2 were highly specific, and complex dynamic behavior such as the ATP concentration dependence of Phase B were largely retained in CP376-RW (Figure 5C). This retention of dynamic behavior may provide further hints to a minimal chaperonin mechanism.

**Figure 9:**
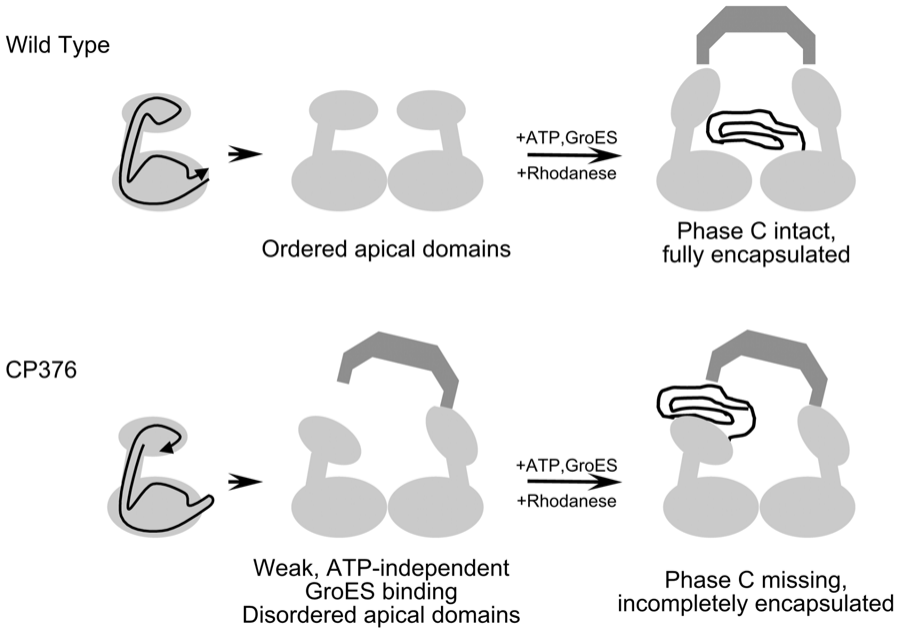
A summary of our findings regarding the CP376 GroEL mutant. For simplicity, only one ring of the GroEL 14-mer is shown. In wild type GroEL (*upper*), the apical domains are arranged about the heptameric ring in an orderly fashion, and through Phase C, act in a coordinated manner to encapsulate and sequester folding intermediates of rhodanese. In CP376 (*lower*) however, this orderly orientation is disrupted, and both static and dynamic characteristics of encapsulation are affected. The dynamic aspects of apical domain disorder were observed by the disappearance of the Phase C kinetic transition in stopped-flow experiments, and the static consequences were reflected in an incomplete encapsulation of refolding rhodanese molecules that resulted in Proteinase K sensitivity of the football complex, as well as an ability to bind the cochaperonin GroES in the absence of ATP. Electron micrographs, CD spectra, and stopped-flow assays suggested that these results were caused by an increased flexibility of the apical domain as a functional unit, rather than by an unfolding of this domain caused by circular permutation (for example, the tryptophan fluorescence of CP376-RW continued to reflect various conformational changes of the GroEL subunit in a manner analogous to the wild type subunit).

We summarize our findings regarding the structural and functional aspects of GroEL CP376 in Figure 9. Our results reflect the consequences of disrupting an extremely coordinated molecular mechanism in GroEL centering on the linkages between the apical and intermediate domains. Nevertheless, GroEL CP376 was capable of improving the refolding yield of rhodanese to a certain extent, via a relatively passive mechanism of aggregation suppression and gradual protein release.

Our use of the circular permutation method should now make possible studies where various molecular interactions are interpreted according to the propagation of structural signals through various segments of the polypeptide backbone of GroEL, in addition to spatial interactions between individual regions of the subunit structure. This possibility should add another interesting viewpoint in the analysis and understanding of the molecular mechanism of the chaperonins.

## Materials and Methods

In this study, all numerical notations used to describe the positions of various CP mutants and point mutations in the amino acid sequence are derived from the numbering of the wild type GroEL amino acid sequence. All molar concentrations denote concentrations of chaperonin oligomer (14-mer for GroEL, 7-mer for GroES).

### Construction of CP GroEL mutants

Circular permutation of the *groEL* gene was performed essentially according to the method developed by Graf and Schachman [15] in their studies on aspartate transcarbamoylase.

#### 1. Preparation of a circular GroEL gene fragment

Starting from the expression vector pUCESL [32], the *groEL* open reading frame was first amplified by PCR using synthesized primers that introduced a *Sal*I site (underlined) to both ends of the gene.

*EL circle (+):* 5′-TAAAGgTcgacGCAGCTAAAGACGTAAAATTCGGTA-3′

*EL circle (-):* 5′-CAtaGgtcgacCATCATGCCGCCCATGCCACCCAT-3′

The *Sal*I sites were introduced at each end so that both the ATG start codon and the TAA stop codon would be overwritten by the restriction enzyme recognition sequence (lower scale letters indicate mismatches to native sequence). The recognition sequence for *Sal*I corresponds to the codons for Val-Asp, and therefore subsequent *Sal*I digestion of the amplified fragment followed by self-ligation into circular DNA fragments (DNA Ligation Kit Mighty Mix, Takara) produced a circular GroEL gene where the original N and C-termini (minus the N-terminal methionine) was connected with a Val-Asp dipeptide linker.

#### 2. Site-specific circular permutation of GroEL (CP209)

This circular GroEL gene fragment was subsequently used to construct linear DNA fragments containing CP GroEL through one of two different methods, outlined as follows. In an initial feasibility study where a single CP GroEL was constructed in a site-directed manner, circular GroEL fragments were purified from agarose gels using the QIAquick gel extraction kit (QIAGEN) and used in PCR reactions as template DNA. The primers used in this experiment were 5′-phosphorylated primers whose 5′ ends corresponded to the amino acid sequence positions 208 and 209 in wild type GroEL (circular permutation mutant CP209).

*ELGlu209(+):* 5′-Pi- GAAACTGGCGCAGTAGAAC-3′

(first three bases correspond to Glu209)

*ELGlu209(-):* 5′-Pi- CGGCTTGTTGATGAAGTAA-3′

(first three bases correspond to Pro208 (reverse complement))

After PCR amplification, the amplified gene fragment was subjected to blunt-end ligation into vector DNA. This vector DNA fragment was obtained by performing a PCR amplification with pUCESL as template, using the following primers that flanked the *groEL* open reading frame and which contained an ATG start codon ("pUCESL-DLlower", underlined, reverse complement) and a "triple-frame-stop codon" ("pUCESL-DL upper", italics; lowercase indicates sequence inserts) [17]:

*pUCESL-DLlower:* 5′-CATTATCTTTATTCCTTAAATTC-3′

*pUCESL-DLupper:* 5′-*TAATTaaataa*CACCTCGCAGAAAT-3′

After ligation of insert and vector, the CP GroEL gene would be sandwiched between the vector-derived start codon and the triple-frame stop codon, and this would allow expression of the gene.

#### 3. Random circular permutation of GroEL and screening for potential candidates (CP254 and CP376)

In subsequent random circular permutation experiments that produced the CP variants CP254 and CP376, aliquots of the original circular GroEL gene fragment were first subjected to a 10 min digestion with DNase I (final concentration: 5×10^−5^ U/ul) in 50 mM Tris-HCl, pH 7.5 and 1 mM MnCl_2_. After digestion, DNA was recovered by phenol-chloroform extraction and ethanol precipitation and next incubated for 1 h at 25°C in the presence of T4 DNA ligase and T4 DNA polymerase to repair various nicks and overhangs caused by the DNase I treatment.

Next, the linearized gene fragments were blunt-end ligated into a pUCESL derived vector that was prepared by PCR amplification, similar to that used in constructing CP209. However, in the random experiments, we used a modified set of pUCESL-DLupper and pUCESL-DLlower primers, where a *Nae*I restriction enzyme site was added to the 5′ end of each primer. After PCR amplification with these modified primers, the linear product was digested with *Nae*I, followed by a brief treatment with calf-intestine alkaline phosphatase to remove terminal phosphates. This additional precaution ensured that the ends of the vector DNA would be consistent, and was added to the protocol because in the initial construction of CP209 we found that small differences (∼1 nt) in the amplified vector DNA fragment, especially in the upstream ATG start codon region, would cause frameshifts and a loss of mutant expression. Partially as a consequence of this precaution, both GroEL CP254 and CP376 contain extraneous amino acids at their N- and C-terminal ends that are not derived from the native amino acid sequence (Table 1, *italics*).

In both the directed mutation experiments and the random experiments, candidate *E. coli* colonies that were obtained after transformation were screened by direct PCR. The reaction was performed using two primers, one of which corresponded to a sequence within *groEL*, while the other corresponded to a sequence derived from the vector. PCR amplification of candidates using these two primers would result in amplified DNA of differing lengths, and by comparing lengths with DNA amplified from wild type template, we were able to rapidly screen prospective candidates. CP254 and CP376 were eventually chosen because of their relatively abundant expression in the supernatant fraction of *E. coli* hosts. DNA sequencing of the candidates was performed to determine the final form of each construct. We found that in CP376, the N-terminal amino acid sequence had been altered slightly, and apparently an insertion of an extra alanine residue had occurred (Table 1, *double underlined*). We decided to name this mutant CP376 based on the fact that the native GroEL amino acid sequence continues uninterrupted after Val376.

#### 4. Optimization of the polypeptide ends of GroEL CP376 and introduction of a tryptophan residue into the apical domain to construct CP376-RW

In order to directly monitor the apical domain movements of GroEL CP376, we introduced a fluorescent tryptophan residue into this mutant at a position corresponding to Arg231 of wild type GroEL [6]. During the construction of this new fluorescent mutant (CP376-RW, Table 1), however, we attempted to improve the overall expression and stability of the protein in *E. coli*. We accomplished this through modification of the N- and C-terminal ends. We prepared a set of PCR primers to amplify the CP376 coding gene, where primers encoded a modified N-terminal with zero, two or four outermost amino acids removed (3 primers) and a C-terminal sequence with zero or two amino acids removed (2 primers; 5 primers in total). By adding these five primers simultaneously and performing PCR amplification of the CP376 gene, we obtained a pool of amplified DNA that contained all 6 possible combinations of N- and C-termini truncations. Amplified DNA was then blunt-end ligated as described above and screened for improved expression in *E. coli.* From the pool we isolated a variant of CP376-RW that lacked two residues from the N-terminal end and the original C-terminus (Table 1), whose overall expression in *E. coli* was greatly improved. This gene fragment was ligated into pET23a(+) to construct an expression plasmid (pETELCP376R231W-del2del0) which was used to transform *E. coli* BL21(DE3). The basal ATPase activity of GroEL CP376-RW was found to be slightly less than that of wild type GroEL and insensitive to GroES (data not shown). Malate dehydrogenase refolding assays revealed that the chaperonin activities of CP376-RW were comparable to GroEL CP376 (data not shown).

The DNA sequences of the four circularly permuted GroEL mutants constructed in this study have been submitted to the GenBank database (accession numbers JN541311 (CP209), JN541312 (CP254), JN541313 (CP376), and JN541314 (CP376-RW)).

### Culture and purification

With the exception of GroEL CP376-RW, circular permutation mutants of GroEL were expressed in *E. coli* JM109 cells cultured in LB medium, pH 7.2, containing 50 µg/ml ampicillin. 0.1% glycerol was added in some cultures to boost cell yields. Cultivation of *E. coli* BL21(DE3)/pETELCP376R231W-del2del0 was performed according to the autoinduction protocol described by Studier [33]. Purifications of each mutant and wild type GroEL were completed according to the protocol described in Machida *et al.* [34]. However, chromatography was performed on an AKTÄ FPLC system (GE Healthcare UK Ltd.) maintained at 4°C. Highly purified samples of GroES were obtained according to the protocol described in Iwasa *et al.* [35], which includes an 80°C heat treatment of GroES samples as initially described by Kamireddi and coworkers [36].

### Functional assays

ATPase activities of wild type and mutant GroEL were measured using the Malachite Green colorimetric assay essentially as outlined in Kubo *et al*. [37]. Data were corrected for spontaneous production of inorganic phosphate that occurred during measurements. Refolding assays of bovine rhodanese (Sigma) were performed under non-permissive conditions according to protocols described previously [38].

### CD spectra

CD spectra were recorded on a JASCO J-820 spectropolarimeter with a temperature-regulated sample holder maintained at 25°C. Spectra were recorded using 0.1 cm quartz cells at a protein concentration of 50 µg/ml in 50 mM Tris-HCl buffer, pH 7.8. The raw signals were converted to mean residue molar ellipticities according to standard equations.

### Stopped-flow fluorescence analysis of GroEL R231W and GroEL CP376-RW

Stopped-flow fluorescence kinetics of GroEL mutants R231W (wild type like) and CP376-RW (circular permutant) were measured on an Applied-Photophysics SX17MV stopped-flow spectrophotometer upgraded to SX20 compatibility with component, flow-line, and software upgrades. Buffer conditions and sample preparation steps were identical to those outlined in Taniguchi *et al.* [6] and Yoshimi *et al.* [21]. The concentration of GroEL during measurements was 0.3 mg/ml and the photovoltmeter voltage was set to 450 V for all traces. The excitation wavelength was 295 nm, and a 320 nm cutoff filter was used to filter the emission spectrum to isolate tryptophan fluorescence changes. Up to 30 individual traces were averaged to obtain the traces shown in Figure 5 A and B and the kinetic constants from these traces were analyzed using KaleidaGraph 4.1 using the double exponential decay equation. To determine the rate constants in Figure 5C, 15 individual traces taken at each ATP concentration were averaged and analyzed using the single exponential decay function on selected portions of the averaged trace.

### Electron microscopy

Electron microscopy of GroEL wild type, GroEL R231W, and GroEL CP376-RW was performed on a JEOL JEM-1210 electron microscope with the voltage set to 85 kV. GroEL was prepared in stopped-flow buffer (50 mM triethanolamine, pH 7.5, containing 20 mM MgCl_2_ and 50 mM KCl) and used for sample preparation. For samples containing ATP and GroES, 0.2 mM ATP and an equimolar concentration of GroES was added to the sample and incubated for 2 h at 37°C to allow exhaustive ATP hydrolysis. Next, 5 µl of each chaperonin sample (GroEL concentration: 0.125 mg/ml for samples containing ATP and GroES, 0.25 mg/ml for samples containing only GroEL) was applied to preformed carbon coated collodion mesh supports (Nissin EM) and incubated for 1 min at room temperature. Excess applied sample was blotted off from the support using filter paper and 5 µl Milli-Q water was applied and then immediately blotted off to wash the surface. A 1% uranyl acetate solution was then applied to the grid, incubated for 20 s, and excess staining solution was again blotted off. After drying these samples were subjected to analysis.

Images were recorded on 8.2×11.8 cm film, developed, and film negatives were digitized using a commercial flatbed scanner (Canon) in 8-bit grayscale film scanning mode.

### Filtration assay to probe the stability of GroEL-ADP-GroES complexes

GroEL R231W and GroEL CP376-RW were incubated in stopped-flow buffer containing an equimolar concentration of GroES for 2 h at 37°C, to mimic conditions used in electron microscopy sample preparation. ATP (0.2 mM) was added where indicated. The concentration of GroEL was 1 mg/ml during incubation. After incubation, 300 µl of each sample was applied to an Amicon Ultra-0.5 100K centrifugal filter unit and concentrated by a 5,000 x g centrifugation for 5 min. Both the concentrate and the filtrate were analyzed on a 15% SDS-PAGE gel to determine the protein composition of each sample after filtration. For filtrate samples, 200 µl of each filtrate was taken and 100% trichloroacetic acid was added to a final concentration of 7% on ice to precipitate protein. Precipitated samples were then resolubilized in SDS sample buffer and applied to the gel in total to obtain the gel profiles denoted "Filt." in Figure 7. For concentrate samples, the protein concentration of each sample was determined and aliquots of the sample were used so that 24 µg protein would be applied to each lane denoted "Conc." in Figure 7. Gels were stained with Coomassie Brilliant Blue.

### Proteinase K resistance assays of fluoroberyllate (BeFx)-stabilized chaperonin football complexes

In order to obtain details regarding the ternary GroEL-GroES-rhodanese complex that is formed in the presence of ATP and BeFx, we performed Proteinase K digestion assays to probe the relative stability of GroEL R231W and CP376-RW football complexes. We used a protocol initially described by Taguchi *et al.* [22], with slight modifications. GroEL R231W and CP376-RW mutants (final 0.5 µM 14-mer, 0.4 mg/ml) were added to rhodanese refolding assay buffer (30 mM Tris-HCl, pH 7.2 at 25°C, containing 50 mM KCl and 10 mM Mg(CH_3_COO)_2_) and then aliquots of unfolded rhodanese from a 5 mg/ml stock solution (incubated for 1 hr in 6 M guanidine hydrochloride (Gdn-HCl) at 25°C) were added to a final concentration of 1.0 µM (33 µg/ml). In samples that omitted unfolded rhodanese, 6 M Gdn-HCl in Milli-Q water was substituted for unfolded rhodanese samples and processed identically. The carry-over residual Gdn-HCl concentration in each sample was 0.04 M. Each mixture was incubated for 10 min at 25°C to allow binding of rhodanese to GroEL; then 1 mM BeCl_2_, 10 mM NaF, 1.1 µM GroES, and 1 mM ATP were added consecutively. After all of the components had been added, samples were incubated at 25°C for an additional 30 min to allow the reaction to stabilize. Samples were then subjected to digestion with 1 µg/ml Proteinase K for 30 min at 25°C. After adding 1 mM PMSF to the reaction to stop digestion, samples were applied to an Amicon Ultra-0.5 100K centrifugal filter unit and concentrated by a 5 min centrifugation at 5,000 x g. An additional 300 µl of rhodanese refolding assay buffer was added to the upper reservoir and this centrifugation step was repeated. Samples concentrated in this manner were quantitated for protein and loaded onto a 15% denaturing polyacrylamide gel for SDS-PAGE analysis. Each lane shown in Figure 8 (excepting the marker lanes) represents approximately 24 µg of final processed sample.
